# The Dark Side of Nrf2 in the Heart

**DOI:** 10.3389/fphys.2020.00722

**Published:** 2020-07-09

**Authors:** Huimei Zang, Roy Oomen Mathew, Taixing Cui

**Affiliations:** ^1^Department of Cell Biology and Anatomy, University of South Carolina School of Medicine, Columbia, SC, United States; ^2^Division of Nephrology, Department of Medicine, Columbia VA Healthcare System, Columbia, SC, United States

**Keywords:** Nrf2, oxidative stress, reductive stress, autophagy, heart failure

## Abstract

Nuclear factor-erythroid factor 2-related factor 2 (Nrf2) is a critical transcription factor that regulates the expression of over 1000 genes in the cell under normal and stressed conditions. These transcripts can be categorized into different groups with distinct functions, including antioxidative defense, detoxification, inflammatory responses, transcription factors, proteasomal and autophagic degradation, and metabolism. Nevertheless, Nrf2 has been historically considered as a crucial regulator of antioxidant defense to protect against various insult-induced organ damage and has evolved as a promising drug target for the treatment of human diseases, such as heart failure. However, burgeoning evidence has revealed a detrimental role of Nrf2 in cardiac pathological remodeling and dysfunction toward heart failure. In this mini-review, we outline recent advances in structural features of Nrf2 and regulation of Nrf2 activity and discuss the emerging dark side of Nrf2 in the heart as well as the potential mechanisms of Nrf2-mediated myocardial damage and dysfunction.

## Introduction

Heart failure is defined as “a complex clinical syndrome that can result from any structural or functional cardiac disorder that impairs the ability of the ventricle to fill with or eject blood” (Hunt et al., 2005; Yancy et al., 2013). Recent studies have revealed that heart failure affects 1 in 5 Americans, and the prevalence and incidence of heart failure are still increasing (Yancy et al., 2013; Virani et al., 2020). Heart failure is usually the last stage of different cardiovascular diseases, such as hypertension that causes sustained pressure overload to the heart; coronary arterial diseases, leading to myocardial infarction or ischemia reperfusion–related cardiac injury; valvular disease, resulting in volume overload to the heart; and congenital heart disease. Despite the differential etiologies, the progression of heart failure goes through a common path; i.e., cardiac remodeling, which has been considered as epigenetic and genomic alterations as well as molecular and cellular responses, resulting in clinically manifested changes in geometry and function of the heart after cardiac pressure or volume overload and/or injury (Swynghedauw, 1999; Cohn et al., 2000; Heusch et al., 2014). Cardiac remodeling may be initially adaptive against various harmful insults but, when sustained, turns out to be maladaptive, or pathological, progressing to structural and functional changes that lead to heart failure. Cardiac maladaptive remodeling is usually characterized by myocardial hypertrophy, fibrosis, and cell death, which may be resulted from a complex interaction between cardiac myocytes and non-myocytes. The molecular mechanism of cardiac maladaptive remodeling that leads to heart failure in diverse pathological settings is poorly understood. The treatment of heart failure remains at a level of controlling symptoms and reducing risk factors without a cure.

Oxidative stress, a state that occurs when the oxidative force, e.g., production of reactive oxygen species (ROS) exceeds the antioxidant capacity, is a common mechanism of various cardiac pathologies leading to heart failure (Tsutsui et al., 2011; Ahmed and Tang, 2012). However, several large clinical trials using antioxidant supplements, such as non-selective ROS scavenger vitamin C, vitamin E, and allopurinol, have found that non-specific scavenging of ROS does not help to prevent cardiovascular disease; instead, it may even be harmful (Li et al., 2009a; Ahmed and Tang, 2012). These studies suggest that effective therapeutic approaches for the treatment of cardiovascular disease may not be achieved without specific targeting the source of oxidative stress or the intrinsic antioxidant system. In this regard, nuclear factor-erythroid factor 2-related factor 2 (Nrf2), which has been historically considered as a master transcription factor of antioxidant defense, has evolved as an attracting therapeutic target for cardiovascular disease (Li et al., 2009a; Chen and Maltagliati, 2018; Ge et al., 2019). Despite the fact that Nrf2 appears to be a critical regulator of cellular defense against various pathological insults in the heart, burgeoning evidence has demonstrated a detrimental role of Nrf2 in cardiac disease progression (Cui et al., 2016). In this mini-review, we update recent advances in structural features and activity regulation of vertebrate Nrf2 and then have a close look on the dual effects of Nrf2 in the heart and discuss the potential molecular mechanisms underlying Nrf2-mediated dichotomy in the heart.

## Nrf2 Signaling

Nrf2-related factor 2 is a member of the Cap “n” Collar (CNC) family of basic leucine zipper (bZip) transcription factors that include nuclear factor-erythroid factor 2 (NF-E2), Nrf1-3, and broad-complex, tramtrack, and bric-a-brac (BTB) and CNC homolog 1 (Bach 1) and Bach 2. The differences between these transcription factors have been broadly reviewed (Li et al., 2009a; Maher and Yamamoto, 2010). Concisely, NF-E2 and Nrf1-3 act as transcriptional activators, whereas Bach 1 and 2 serve as transcriptional repressors. The expression of Nrf proteins is ubiquitous in the body. Nrf1 plays a role in controlling the basal gene expression level of some cytoprotective enzymes but does not regulate their inducible expression. Although marginal, Nrf3 is capable of regulating the gene expression of phase 2 enzymes. In contrast, Nrf2 binds to a *cis*-acting enhancer with a core nucleotide sequence of 5′-RTGACNNNGC-3′, that is also known as the antioxidant response element (ARE), or the electrophile response element (EpRE), to control the basal and inducible expression of over 1000 genes that can be clustered into several groups with distinct functions, including antioxidative defense, detoxification, inflammatory responses, transcription factors, proteasomal and autophagic degradation, and metabolism (Hayes and Dinkova-Kostova, 2014; Cui et al., 2016; Kopacz et al., 2020). Thus, Nrf2 is multifunctional with cellular functions ranging from antioxidative defense to protein quality control to metabolism regulation.

**FIGURE 1:**
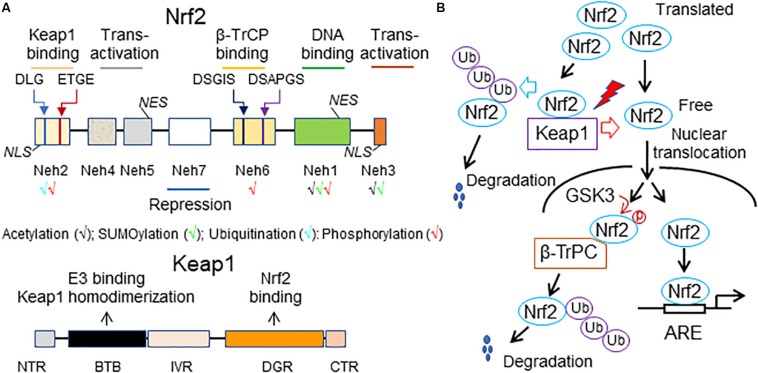
A working model of Nrf2 signaling. **(A)** Structural features of Nrf2 and Keap1. The high-affinity ETGE and low-affinity DLG motifs in the Neh2 domain of Nrf2 are bound by the Kelch domain of Keap1 for Nrf2 ubiquitination and degradation. Neh, Nrf2-erythroid-derived CNC homology (ECH) domain; NES, nuclear export signal; β-TrCP, the dimeric β–transducin repeat-containing protein; NLS, nuclear localization signal; BTB, broad-complex, tramtrack and bric-a-brac domain; DGR or Kelch, double glycine repeat domain; NTR, the N-terminal region; IVR, intervening region; CTR, the carboxyl terminal region. **(B)** A scheme of Nrf2 activity regulation. Under homeostatic conditions, translated Nrf2 binds to Keap1, leading to ubiquitination of Nrf2 and subsequent proteasomal degradation of the ubiquitinated Nrf2. As a result, there is only a small amount of free Nrf2 translocating into the nucleus and activating the basal expression of Nrf2 target genes. Under stressed conditions, various stressors (red lighting symbol) interrupt the interaction of Keap1 with Nrf2, thus resulting in decreases in proteasomal degradation of Nrf2 and increases in *de novo* Nrf2 free of Keap1 binding. Accordingly, the nuclear translocation of Nrf2 and subsequent Nrf2-driven transcription are increased, thereby intensifying Nrf2-mediated defense against the stress-induced imbalance of redox status and damage in the cell. An alternative mechanism of proteasomal degradation of Nrf2 independent of Keap1 is mediated by glycogen synthase kinase 3 (GSK3) and β-TrCP. Under normal conditions, GSK3 is maintained in an inactive state due to its inhibition by AKT-mediated phosphorylation at its N-terminal pseudosubstrate domain. However, once AKT is inactivated, GSK3 phosphorylates Nrf2 at the Neh6 domain. This phosphorylation recruits β-TrCP and initiates β-TrCP-mediated proteasomal degradation of Nrf2. Ub, ubiquitin.

Structural features of vertebrate Nrf2 have been revised and updated in detail (Hayes and Dinkova-Kostova, 2014; Silva-Islas and Maldonado, 2018; Kopacz et al., 2020). Briefly, Nrf2 has 7 Nrf2-erythroid-derived CNC homology (ECH; Neh) domains (Neh1-7), which are critical for its activity or its repression (Figure 1A). The amino-terminal Neh2 via the DLG and ETGE motifs binds the double glycine repeat (DGR) domain of Kelch-like ECH associating protein 1 (Keap1), a negative regulator of Nrf2. A nuclear localization signal (NLS) sequence is localized in this domain. The Neh6 is another degron region, a portion of a protein important for its degradation, which via the DSGIS and DSAPGS motifs, recruits the dimeric β–transducin repeat-containing protein (β-TrCP), a substrate adaptor for the S-phase kinase 1 (Skp1)-Cullin 1 (Cul1)-Ring box protein 1 (Rbx1) core E3 ligase (i.e., SCF^β–TrCP^). The Neh6 is important for Nrf2 degradation in stressed cells independent of Keap1. The carboxyl-terminal Neh3 is necessary for transcriptional activation of Nrf2 by recruiting coactivator, chromo-ATPase/helicase DNA-binding protein (CDH) 6. The Neh3 contains a second NLS sequence. The Neh1 adjacent to the Neh3 contains a bZip structure, which is critical for DNA binding and dimerization with other transcription factors. A nuclear export signal (NES) sequence is localized in the Neh1. The Neh4 and Neh5 are two independent transactivation domains that interact with cAMP response element-binding protein (CREB)-binding protein (CBP) and/or receptor-associated coactivator 3 (RAC3). The Neh7 mediates repression of Nrf2 by physical interaction with retinoid X receptor alpha (RXRα).

Nrf2-related factor 2 is a short-lived protein with a half-life less than 20 min in the cell (Li et al., 2009a; Chen and Maltagliati, 2018). The expression and activity of Nrf2 are tightly regulated at multiple levels, which have been recently reviewed (Hayes and Dinkova-Kostova, 2014; Chen and Maltagliati, 2018; Silva-Islas and Maldonado, 2018; Kopacz et al., 2020). Generally, the transcription of Nrf2 is activated by itself and other transcription factors, including the aryl hydrocarbon receptor (AhR), peroxisome proliferator-activated receptor (PPAR)α or PPARγ, nuclear factor (NF)-κB (NF-κB), specificity protein 1 (Sp-1), p53, myocyte-specific enhancer factor 2 D (MEF2D), c-Jun, c-Myc, and breast cancer 1 (BRCA1). Epigenetic regulations, such as methylation of the Nrf2 promoter in CpG islands or H3 histone and acetylation of H4 histone, are also involved in Nrf2 transcriptional control. Moreover, Nrf2 synthesis can be downregulated by several miRNAs, including miR27a, miR-28, miR-34a, miR-93, miR-129-5p, miR-142-5p, miR-144, miR-153, miR-155, miR-200c, miR-340-5p, miR-350a, miR-507, and miR-634 at the posttranscriptional level. Nevertheless, the protein stability and transcriptional activity of Nrf2 are mainly regulated by Keap1. Keap1 contains two major domains, BTB and DGR or Kelch, and three additional domains, the N-terminal region (NTR), the intervening region (IVR), and CTR (Figure 1A). The BTB is critical for Keap1 homodimerization and interaction with Cul3-Rbx1-E3 ligase complex while the Kelch binds to the DLG and ETGE motifs in Neh2 of Nrf2. Keap1 contains many cysteine residues sensing oxidative and/or electrophilic molecules in both BTB and IVR. Normally, Keap1 constantly targets Nrf2 for degradation. The current diagram of Keap1 and Nrf2 interaction for Nrf2 degradation is the “hinge and latch” model: The ETGE motif of Nrf2 acts as the “hinge” while the DIG motif of Nrf2 functions as the “latch.” Nrf2 sequentially binds to first one of the Keap1 homodimers via the ETGE motif to form an “open” conformation, prior to the DLG motif being captured by the other Keap1 to form a “closed” conformation that enables Nrf2 ubiquitination by Clu3-Rbx1-E3 ligase for proteasomal degradation and subsequently release of free Keap1. The free or regenerated Keap1 bind to newly synthesized Nrf2 to start another cycle of the Nrf2 degradation. Only a small and steady amount of Nrf2 that is not sequestered by Keap1 for degradation translocate into the nucleus, contributing to the basal expression of ARE-driven genes (Figure 1B). Under stressed conditions, the stressors that are usually oxidative or electrophilic molecules react with the cysteine residues of Keap1 to cause conformational changes in Keap1, leading to downregulation of Keap1-mediated ubiquitination and degradation of Nrf2. However, such modulation of Keap1 does not result in release of Nrf2; instead, it stabilizes the Keap1-Nrf2 interaction that blocks Nrf2 ubiquitination, thereby saturating the cellular pool of the Keap1-E3 complex. As a result, *de novo* Nrf2 free of Keap1 binding that translocates into the nucleus is increased, thereby enhancing the transcription of Nrf2-driven genes. However, there is an alternative mechanism for proteasomal degradation of Nrf2 independent of Keap1, which is mediated by glycogen synthase kinase 3 (GSK3) and β-TrCP (Figure 1B). Under normal conditions, GSK3 is maintained in an inactive state due to its inhibition by AKT-mediated phosphorylation at its N-terminal pseudosubstrate domain. However, once AKT is inactive, GSK3 phosphorylates Nrf2 at the Neh6. This phosphorylation recruits β-TrCP and initiates β-TrCP-mediated proteasomal degradation of Nrf2. Usually, Keap1-mediated proteasomal degradation of Nrf2 occurs in the cytosol, whereas GSK3-mediated proteasomal degradation of Nrf2 happens in the nucleus. The relative importance of Keap1 and GSK3-β-TrCP in controlling the magnitude and duration of Nrf2 activation remains poorly understood.

It should be noted that not only GSK3, but also other kinases, such as AMP-activated protein kinase (AMPK) and mechanistic target of rapamycin complex 1 (mTORC1), directly or indirectly regulate Nrf2 stability and activity (Hayes and Dinkova-Kostova, 2014; Silva-Islas and Maldonado, 2018). In addition, ubiquitination of Nrf2 at the Neh2 can suppress Nrf2 activity, whereas acetylation of Nrf2 at the Neh1 and Neh3 may activate Nrf2 activity. SUMOylation of Nrf2 at the Neh1 and Neh3 may result in either Nrf2 activation or Nrf2 repression (Hayes and Dinkova-Kostova, 2014; Silva-Islas and Maldonado, 2018). Moreover, not only oxidative and electrophilic reactions, but also other types of posttranslational modifications of Keap1, such as ubiquitination and phosphorylation, appear to be critical for regulating Nrf2 activity, which has been comprehensively discussed in a recent review (Kopacz et al., 2020). However, the pathophysiological relevance of these findings in the heart remains to be investigated.

## Nrf2-Mediated Cardiac Protection

Zhu et al. (2008) demonstrated for the first time an Nrf2-dependent cytoprotection against oxidative and electrophilic stress in cardiomyocytes using neonatal mouse ventricular myocytes of Nrf2 knockout (KO) mice. He et al. subsequently documented that KO of Nrf2 enhances ROS production and exaggerates cell death in cultured adult cardiomyocytes in a setting of high glucose-induced oxidative stress (He et al., 2009). While Sussan et al. (2009) found that global KO of Nrf2 enhances cigarette smoke–induced cardiac dysfunction in mice, we further demonstrated that the loss of Nrf2 function accelerates the transition from cardiac compensatory adaptation to heart failure in a setting of pressure overload (Li et al., 2009b). As we demonstrated that pharmacological activation of Nrf2 suppresses oxidative stress-dependent death in cardiac myocytes (Ichikawa et al., 2009; Li et al., 2010), Zhang et al. (2010) documented that Nrf2-deficient cardiomyocytes are more susceptible to 4-hydroxy-2-nonenal (4-HNE) challenge, and the cardiac protection of 4-HNE pre-conditioning is dependent on Nrf2-operated antioxidant defense. Ashrafian et al. (2012) also showed that fumarate-induced suppression of ischemia-reperfusion myocardial injury is wiped out in global Nrf2 KO mice, and Katsumata et al. revealed that Nrf2 is also essential for prostaglandin D2 (PGD_2_)-mediated cardiac protection against ischemia-reperfusion injury in mice (Katsumata et al., 2014). We further demonstrated that conventionally cardiomyocyte-restricted (CR) transgenic overexpression of Nrf2 protects against myocardial oxidative stress, cell death, fibrosis, hypertrophy, and dysfunction in a setting of sustained pressure overload induced by 4 weeks of transverse aortic arch constriction (TAC) in mice (Wang et al., 2014). Moreover, He et al. reported that global KO of Nrf2 could exaggerate cardiac oxidative stress, fibrosis, and apoptosis; contractility of cardiomyocytes; and death within 2 weeks after onset of type 1 diabetes induced by a single i.p. injection of 150 mg/kg streptozotocin (STZ) in mice (He and Ma, 2012), and Gu et al. (2017) showed similar phenotypes in Nrf2 KO mice associated with type 2 diabetes, that is induced by 7 months of a high fat diet (HFD) containing 60% kcal fat with a single i.p. injection of 100 mg/kg STZ at 3 months. Collectively, these findings clearly demonstrate a cardioprotective role of Nrf2 in various pathological settings. Other studies regarding Nrf2-mediated cardiac protection and potential underlying mechanisms have been recently reviewed (Cui et al., 2016; Chen and Maltagliati, 2018; da Costa et al., 2019; Ge et al., 2019). Mechanistically, Nrf2 may activate antioxidant defense, regulate metabolism, and control autophagy and proteasome function, thereby contributing to cardiac protection.

## Nrf2-Mediated Cardiac Damage

A Nrf2-mediated myocardial injury was first observed by Kannan et al. (2013) in aging cardiomyocyte-restricted human mutant CryAB transgenic (CR-hCryAB Tg) mice. Of note, CR-hCryAB overexpression-induced death and cardiac dysfunction associated with aging were dramatically rescued by global KO of Nrf2. At the molecular level, the persistent activation of Nrf2-driven antioxidant gene expression toward a reductive stress has been proposed as a contributing mechanism to CR-hCryAB Tg-induced cardiomyopathy (Kannan et al., 2013). While Allwood et al. found that CR transgenic overexpression of Ho1, an established downstream gene of Nrf2 in the heart (Li et al., 2009b), results in spontaneous development of heart failure at age of 1 year and exacerbates pressure overload-induced cardiomyopathy in mice (Allwood et al., 2014), we demonstrated that pathophysiological consequences of Nrf2 activation are linked to the functional integrity of autophagy in pressure overloaded mouse hearts (Qin et al., 2016). We have established that pressure overload via TAC initially results in an adaptive cardiac hypertrophy with preserved cardiac function (weeks 1–2) followed by maladaptive cardiac remodeling and dysfunction (weeks 2–4), which eventually causes heart failure in wild-type mice (Li et al., 2009b). Using this TAC model, we found that TAC-induced myocardial necrosis and death rate are increased in Nrf2 KO mice in a C57BL/6J genetic background within first 2 weeks (Qin et al., 2016). These results underscore a critical role of Nrf2 in mediating cardiac protection during the initial stage of pressure overload-induced cardiac adaptation. However, we found unexpectedly that Nrf2 KO attenuates cardiac hypertrophy and ameliorates progression of cardiac dysfunction by 8 weeks after TAC (Qin et al., 2016). These results reveal a mediator role of Nrf2 in pressure overload–induced cardiac maladaptive remodeling and dysfunction. A time course study of autophagy functional states in wild-type C57BL/6J mice showed that myocardial autophagy flux is intact at 2 weeks, suppressed at 4 weeks, and blocked at 8 weeks in the hearts after TAC (Qin et al., 2016). Since Nrf2 KO diminishes cardiac adaptation and leads to cardiac dysfunction at 2 weeks after TAC (Li et al., 2009b), when cardiac autophagy flux remains normal, it is most likely that Nrf2 activation is cardioprotective in pressure overloaded hearts when myocardial autophagy function is intact. Given that the Nrf2-mediated cardiac pathological hypertrophy and dysfunction are associated with impaired autophagy in the heart, it is conceivable that Nrf2 activation is detrimental to autophagy-impaired hearts. Indeed, genetic inhibition of autophagy, such as CR-Atg5 KO, in combination with pharmacological kinase inhibitors, demonstrated that cardiac autophagy inhibition activates Fyn-operating Nrf2 nuclear export for degradation, thus enhancing Nrf2-driven transcription of angiotensinogen in cardiomyocytes, thereby leading to pathological activation of renin-angiotensin system in pressure overloaded hearts. These results are seemly contradictory to our findings that Nrf2 activation enhances autophagosome formation and autophagic degradation of protein aggregates, thereby protecting against 4-week TAC-induced cardiac maladaptive remodeling and dysfunction in FVB/N mice (Wang et al., 2014). However, it should be noted that Nrf2 does not regulate the expression of any autophagy-related genes, suggesting that Nrf2 may not directly activate autophagy but indirectly facilitates autophagy activation and degradation in cardiomyocytes (Wang et al., 2014). Given that genetic backgrounds have great impact on autophagy regulation in the heart (Moulis and Vindis, 2017), TAC induces myocardial autophagy inhibition at 4 weeks in C57BL/6J mice (Qin et al., 2016), but it may not induce the same phenotype at 4 weeks in FVB/N mice. Although this notion remains to be clarified, previous studies have shown that the basal level of myocardial autophagy is much higher in FVB/N mice (∼1-fold increases in autophagic flux by 6 h of chloroquine at a dose of 50 mg/kg, i.p.) compared to C57BL/6J mice (∼0.5-fold increases in autophagic flux by 6 h of chloroquine at a dose of 50 mg/kg, i.p.; Fernandez et al., 2018; Deng et al., 2020). Thus, a plausible explanation is that CR-Nrf2 Tg overexpression-induced cardiac protection is mostly likely due to an intact state of myocardial autophagy by 4 weeks after TAC in FVB/N mice. Collectively, our findings suggest that autophagy impairment switches on Nrf2-mediated cardiac pathological remodeling and dysfunction. Interestingly, Bhide et al. (2018) documented that in a Drosophila model of laminopathy, laminopathy-associated age-dependent cardiac dysfunction, could be rescued by knockdown of Nrf2, or enhancement of autophagy in the heart. These findings suggest that age-dependent autophagy deficiency may turn on Nrf2-mediated cardiac dysfunction in the Drosophila model of laminopathy. On the other hand, Erkens et al. (2018) reported that global KO of Nrf2 attenuates myocardial ischemia-reperfusion injury and dysfunction most likely due to an increase in cardiac nitric oxide (NO) production in mice, revealing a detrimental effect of Nrf2-mediated suppression of NO production to the heart. Taken together, these genetic studies have clearly demonstrated a detrimental role of Nrf2 in the heart and Nrf2-mediated myocardial damage is likely occurring during the disease progression (Table 1). Since cardiac function is normal in CR-Nrf2 Tg mice at the age of ∼3 months (Wang et al., 2014), it is highly possible that additional factors are required to turn on the Nrf2-mediated reductive stress causing cardiomyopathy. Although the precise mechanisms activating Nrf2-mediated cardiac damage are unclear, myocardial autophagy inhibition may be one of the critical triggers. However, the downstream signaling of Nrf2-mediated myocardial damage remains to be dissected.

**TABLE 1:** Nrf2 signaling cascade in mediating cardiac damage and dysfunction.

Study	Nrf2 signaling	Animal model	Pathological setting	Intervention	Phenotype	Proposed mechanism
Kannan et al., 2013	Nrf2	CR-hCryAB Tg and global Nrf2 KO in C57BL/6J mice	Aging	None	CR-hCryAB Tg-induced cardiac accumulation of protein aggregates and reductive stress, cardiomyopathy, and heart failure are rescued by additional global KO of Nrf2	Nrf2-mediated reductive stress in the heart
Allwood et al., 2014	HO-1	CR-Ho1 Tg in FVB mice	Aging, Pressure overload (PO), Excess beta-adrenergic activity (Isoproterenol infusion),	None	CR-Ho1 Tg mice develop spontaneous heart failure at age of 1 year, and exacerbated cardiac dysfunction induced by PO	HO-1-mediated loss of adaptive angiogenesis
Qin et al., 2016	Nrf2	Global Nrf2 KO in C57BL/6J mice	Pressure overload (PO)	None	Sustained PO leads to cardiac autophagy impairment and Nrf2 activation; Nrf2 KO attenuates progression of cardiac pathological remodeling and dysfunction in PO hearts.	Sustained PO leads to cardiac autophagy impairment, which in turn activates Nrf2-driven angiotensinogen expression, thereby contributing to progression of cardiac pathological remodeling and dysfunction
Erkens et al., 2018	Nrf2	Global Nrf2 KO in C57BL/6J mice	30 min myocardial ischemia followed by 24 h of reperfusion *in vivo*	Infusion of NOS inhibitor S-ethylisothiourea hydrobromide (ETU)	Nrf2KO attenuates myocardial ischemia/reperfusion injury and dysfunction	Nrf2KO upregulates cardiac NO
Bhide et al., 2018	Nrf2	CR-LamC-R205W and G489V Tg and CncC (Nrf2) RNAi in Drosophila	Aging, Laminopathy	None	CR-LamC mutant Tg-induced age-dependent cardiac dysfunction is rescued by knockdown of Nrf2	Autophagy impairment; Nrf2 persistent activation leading to disruption of redox homeostasis, defective mitochondria, dysregulation of energy homeostasis and energy sensor

## Final Remark

It is evident that Nrf2 could either protect against or exacerbate cardiac damage and dysfunction depending on the pathological nature of disease settings. Our data shows that Nrf2 is crucial for cardiac adaptation when cardiac autophagy is normal while exaggerating cardiac pathological decompensation when myocardial autophagy is impaired in pressure-overloaded hearts (Qin et al., 2016). Notably, autophagy inhibition is a contributory mechanism of protein aggregate-induced cardiomyopathies in aging CR-hCryAB Tg mice (Bhuiyan et al., 2013), sustained pressure overloaded mice (Qin et al., 2016), myocardial ischemia-reperfusion mice (Ma et al., 2012), and aging Drosophila with laminopathy (Bhide et al., 2018). Therefore, it is likely that autophagy inhibition is essential for activating Nrf2-mediated cardiac damage toward heart failure. These results raise a concern regarding the potential activation of Nrf2-mediated cardiac damage in the clinical therapies when treated subjects are compounded with diabetic, hypertensive, and ischemic cardiomyopathies, all of which likely have myocardial autophagy inhibition (Wang and Cui, 2017).

Notably, the clinical phase III trial testing the therapeutic effect of Bardoxolone methyl, a potent Nrf2 activator, on chronic renal disease associated with type 2 diabetes was terminated because of an increased rate of cardiovascular events, including heart failure and deaths (de Zeeuw et al., 2013). The underlying mechanism remains to be determined. Given that autophagy inhibition also occurs in diabetic hearts (Ouyang et al., 2014; Kobayashi and Liang, 2015), it is intriguing whether the “dark” side of Nrf2 activation contributes to the failure of the Bardoxolone methyl clinical trial. Nevertheless, the enthusiasm for activating Nrf2 as a novel approach to treat human disease, at least non-cardiac diseases, remains very high (Al-Sawaf et al., 2015; Robledinos-Anton et al., 2019). Several clinical trials of a few Nrf2 activators for treating other types of disease, including Bardoxolone methyl, Omaveloxolone, dimethyl fumarate, ALKS-8700 (a fumarate acid ester), Oltipraz, Ursodiol, Sulforaphane, Sulforadex, and Curcumin are still actively ongoing (Robledinos-Anton et al., 2019). All these pharmacological Nrf2 activators are electrophilic compounds that could covalently modify cysteine residues in Keap1 by oxidation or alkylation to cause conformational changes in Keap1, leading to inhibition of Keap1-mediated degradation of Nrf2, thus increasing the amount of newly synthesized and free Nrf2 and consequent enhancement of Nrf2-operating transcription (Robledinos-Anton et al., 2019). Thus, these so-called “Nrf2 activators” are actually “Keap1 inhibitors.” Recent studies have revealed that Keap1 is not limited to control Nrf2 activity, but also required for S-nitrosation, proteostasis, mitochondria homeostasis, cytoskeleton regulation, and cell cycle progression (Kopacz et al., 2020). However, the impact of these Nrf2 activators or Keap1 inhibitors on the Keap1-mediated actions beyond inactivation of Nrf2 remains largely unknown.

Taken together, further investigation of molecular mechanisms of Nrf2-mediated myocardial damage, such as autophagy-mediated control of Nrf2 signaling in the heart, will lead to a better understanding of Nrf2-medicated dichotomy in the pathogenesis of cardiomyopathies toward heart failure. The off-target effects of Nrf2 activating compounds (Keap1 inhibitors) in the heart, particularly the possibility of interrupting Keap1 functions independent of Nrf2 degradation, have to be characterized. As a result, the outcome will provide novel insight into the development of new effective approaches to target Nrf2 signaling for the treatment of cardiac and non-cardiac diseases.

## Author Contributions

HZ and RM wrote the draft. TC provided research funds and finalized the manuscript. All authors contributed to the article and approved the submitted version.

## Conflict of Interest

The authors declare that the research was conducted in the absence of any commercial or financial relationships that could be construed as a potential conflict of interest.
